# A study on the examination of sense of coherence-related factors in Japanese junior high school students and their mothers

**DOI:** 10.1038/s41598-022-07998-4

**Published:** 2022-03-10

**Authors:** Tomoko Omiya, Naoko Kumada Deguchi, Taisuke Togari, Yoshihiko Yamazaki

**Affiliations:** 1 Division of Health Innovation and Nursing, Department of Public Health Nursing, Faculty of Medicine, University of Tsukuba, 1-1-1 Tennnodai, Tsukuba-city, Ibaraki Prefecture 305-8575 Japan; 2 Faculty of Liberal Arts, The Open University of Japan, Chiba-city, Chiba Prefecture Japan; 3 Department of Social Welfare, Faculty of Social Welfare, Nihon Fukushi University, Chita-Gun, Aichi Prefecture Japan

**Keywords:** Psychology, Health care

## Abstract

This study examined the relationship between the sense of coherence (SOC), which is conceptually the ability to successfully cope with stress, of Japanese junior high school students and their mothers, and investigated SOC-related factors among the students. We analyzed self-reported data from 134 junior high school students (aged 12–14 years) and their mothers (average age: 45.5 ± 4.1 years), based in an urban area of Japan. We found a weak correlation between the SOC total scores of female students and their mothers; further, few subscales showed weak correlations. However, the SOC of male students was not correlated with that of their mothers, including the three subcomponents that constitute the SOC. The results of multiple regression analysis indicated that the SOC of female students was negatively associated with ASD-related characteristics, sensitivity to evaluation, and avoidant help-seeking style, and positively associated with a sense of belonging to the school. However, male students’ SOC showed a negative relationship with excessive response to evaluation and a strong sense of parental control. To improve SOC in adolescents, it is necessary to consider their gender differences and support them, both at home and school.

## Introduction

Sense of coherence (SOC) was originally conceptualized by Antonovsky^1^, as the ability to protect physical and mental health and succeed in coping with even severe stressors in our living world. SOC is at the core of salutogenesis, which aims to elucidate, support, and strengthen salutary factors by considering how health is recovered, maintained, and enhanced. SOC can also help transform stress into a source of growth and development. According to Antonovsky^2^, individuals with a strong SOC are less likely to perceive everyday situations as being stressful. Put differently, a SOC helps agents select appropriate coping strategies more frequently, and consequently, experience fewer detrimental health effects associated with stress^3^. Conceptually, a SOC comprises three components: comprehensibility, manageability, and meaningfulness. Comprehensibility refers to the extent one can perceive internal and external stimuli as understandable. Meaningfulness implies that a person is engaged with something in life that is important to them on both an emotional and a cognitive level. Manageability focuses on available resources needed to meet an individual’s demands^2^. Previous studies indicate that factors such as a high economic status^4^, high educational background^5^, and stable family relationships, are all associated with a strong SOC^6^. Moreover, in recent years, it has been a topic of great research interest, primarily in Europe and the US, as well as in Japan^7,8^.

It is a predictor of depression and adjustment disorders^9,10^, and studies have shown that a strong SOC provides psychological stability to adolescents, and helps them adjust to the school environment and society^11^. However, Antonovsky^1,12^ stated that SOC in adolescents is very vulnerable, and suggested that receiving trust and affection from teachers, friends, and family has a significant impact on the development thereof in adolescents. Antonovsky^1^ stated that family relationships are associated with SOC formation, including the possibility that involvement in decisionmaking at home may foster a SOC in children; however, he did not clearly indicate if a SOC is passed down in families as an inherited trait. A study by Omiya et al. showed that the SOC of high school female students and their mothers is correlated and that the behaviors of the mothers and female students in families may influence each other^13^. Clarifying the association and influence of a SOC among family members in vulnerable adolescents, may help elucidate how a SOC is nurtured in children. Additionally, clarifying these issues can help advise parents and children on appropriate behaviors at home to foster a strong SOC in children. To our knowledge, no study has examined the association between SOC in junior high school students and their parents.

School is an integral part of an adolescent’s life; close friendships play a major role in adolescence and significantly affect an individual’s school life. The psychological characteristics associated with this developmental stage can increase one’s sensitivity to evaluation by others. In a previous study on adolescents, many participants reported experiencing social intimidation, making statements such as “I wonder what people think of me,” “I feel tense when interacting with people,” and “I cannot fit in groups”^14^. Ishizu and Ambo^15^ also pointed out that, while children who are sensitive to others’ evaluation have high social skills and appear to be adapted to school, they are also likely to be over-adapted. The tendency for over-adaptation may be reflected in a high-achiever student who understands the intentions of others, and at the same time, shows a high tendency toward depression^16^. In such a case, even if there is a tendency toward depression, parents and teachers judge a student as adapted and find it difficult to recognize the child’s help-seeking behavior. It has also been observed that model students are hesitant to request help^15^. Therefore, it is necessary to examine the relationship between adolescents’ susceptibility to others’ evaluation, decreased mental health, and help-seeking attitudes.

In school, students develop close relationships with each other through regular communication. They cannot develop friendships if they find it difficult to understand others’ intentions or have unsuccessful interactions; such experiences can lead to school refusal, depression, and bullying. In a study on high school students, Omiya et al. found that high levels of the communication-related characteristics of autism spectrum disorder (ASD) were associated with a weak SOC in male students^13^. Since the characteristics of ASD are distributed on a continuum, the concept of a spectrum has been proposed^17^. This indicates that, while students with significantly high ASD characteristics receive a diagnosis, there are many children with ASD tendencies in regular classes, who do not^18^. In Japan, there are many cases where ASD is not diagnosed in childhood, and depression or adjustment disorder appears as a secondary disorder in adulthood^19,20^. Since a SOC is a predictor of depression and adjustment disorder^9,10^, it is necessary to examine the relationship between SOC and ASD in junior high school students, from the perspective of depression and suicide prevention; however, thus far, no study has examined ASD tendencies and a SOC in junior high school students attending regular classes.

This study aimed to examine the relationship between a SOC of adolescents and their mothers, and to identify factors associated therewith in adolescents who are experiencing stress and difficulties at school. The participants were Japanese junior high school students and their mothers. To gain insight into the process of a SOC formation, we examined how the SOC of junior high school students was related to that of their mothers. We also examined the relationship between junior high school students' SOC and school- and family-related variables, personality tendencies and traits, and parenting styles.

### Research questions

- Based on Antonovsky’s theory, is there a correlation between the SOC of junior high school students (early adolescents) and the SOC of their mothers?
- What are the factors related to the SOC of Japanese junior high school students?

### Hypothesis

- The SOC of female junior high school students is correlated to their mothers’ SOC.
- Junior high school students’ SOC is related to their own personality traits (interpersonal sensitivity, ASD-related traits, and help-seeking behavior), family factors (subjective parenting styles), and the way they perceive school life.

## Method

### Sample and data collection

The participants were 320 first- and second-year students attending a junior high school in Tokyo, Japan (age range: 12–14 years) and their mothers. In Japan, the responsibilities of childcare fall heavily on mothers, as it has long been the social norm for fathers to focus primarily on work and spend long hours away from home^21^. Thus, research in Japan suggests that adolescents’ relationships with their mothers may significantly impact their emotional growth and development^22^. Consequently, in this study, we focused only on mothers.

Data were collected using an anonymous self-administered questionnaire survey, between January and March 2018. The questionnaires were distributed to the students by their class teachers and the students took home the questionnaires for their mothers. Students were asked to submit completed questionnaires using a collection box placed in their school, while the mothers were asked to return their questionnaires by mail. We received responses from 158 students, with a response rate of 49.4%. Responses from students were analyzed only if their mothers also responded to the questionnaire. Overall, 134 mothers (41.9%) returned completed questionnaires. Additionally, among the respondents, there were no cases where a mother had two children in the same school.

### Ethical considerations

This study complied with the rules of the Declaration of Helsinki and was conducted with the approval of the medical ethics review board of the University of Tsukuba (approval No. 1251; November 30, 2017). In accordance with ethical guidelines, we obtained written consent from each student’s parents regarding their participation in the survey, since the present sample comprised junior high school students. We explained that participation in the survey was voluntary and that withdrawing participation at any time would not be penalized. We distributed the questionnaire only to students who provided a handwritten signature from their parents to confirm their intention to participate. We set up a response section in each questionnaire for both the students and mothers to confirm their intention to participate and checked whether there was a statement of intention to participate. We also assigned a common number to each mother-student pair to conduct a joint analysis; however, we explained in writing that there was no match between the students’ names and their assigned numbers, thereby maintaining anonymity.

### Variables and questionnaires

Each student’s gender and age were recorded as basic demographic attributes. The students’ mothers were asked to provide their age, educational background, financial status, occupation, and marital status.

#### Measures for both mothers and adolescents

##### Sense of coherence (SOC)

To measure SOC, we used the Japanese version of the Sense of Coherence 13-item Scale (SOC-13), which is a shortened version of Antonovsky’s original 29-item scale. The SOC-13 was developed by Togari and Yamazaki^23^, and validated through a nationwide survey in Japan. In the original 29-item scale, responses are provided on a 7-point scale; however, in the SOC-13, the items are rated on a 5-point scale. Additionally, the SOC-13 contains three subscales: comprehensibility (5 items), manageability (4 items), and meaningfulness (4 items). Higher scores indicate higher health-generating and stress-coping ability. In this study, Cronbach’s alphas of the SOC-13 among students and mothers, were 0.835 and 0.860, respectively.

#### Measurements for adolescents

##### School membership scale

To measure the students’ sense of school membership, we used the Japanese short version of the School Membership Scale, adapted from Goodenow’s Psychological Sense of School Membership Scale^24^. This scale has three components: acceptance by students (five items), acceptance by teachers (four items), and sense of belonging (four items). The reliability and validity of the Japanese version have been previously confirmed^25^. Responses are provided on a 5-point Likert scale and summed to obtain a total score. In this study, Cronbach’s alpha for this scale was 0.790.

##### Help-seeking styles

We used a scale developed and validated by Nagai^26^, that measures two types of help-seeking styles: excessive help-seeking (seeking more help than is necessary) and avoidant help-seeking (rarely seeking help). The scale comprises eight items rated on a 7-point Likert scale. Scores are summed up to provide a total score. In this study, Cronbach’s alphas were 0.914 and 0.927, for excessive and avoidant help-seeking, respectively.

##### Parenting in adolescence scale (PAS)

Utsumi^27^ developed and validated the PAS, based on the Children’s Report on Parental Behavior^28^. The scale assesses parenting behavior by examining children’s perspectives. It comprises three subscales: acceptance (which relates to emotional support and perception of warmth; six items), excessive psychological parental control (which is concerned with controlling the child’s behavior by intervening in the child’s emotions and thoughts; six items), and monitoring (which relates to tracking and paying attention to the child’s current location, as well as the child’s activities and adaptations; three items). Items are scored using a 7-point Likert scale, with responses ranging from 1 (“not applicable at all”) to 7 (“very applicable”), and a total score is calculated by summing up all items. In the present study, Cronbach’s alphas for the three subscales were 0.915, 0.826, and 0.709, respectively.

##### Interpersonal sensitivity scale

We used the interpersonal sensitivity subscale of the Interpersonal Sensitivity/Privileged Self Scale, developed by Muranaka et al.^29^. This subscale assesses sensitivity to evaluation (caring about others’ opinions) and excessive response to evaluation (being sensitive to the words of others and being shocked and hurt by minor criticism), both of which are measured by seven items each, scored on a 5-point Likert scale. In this study, Cronbach’s alphas for sensitivity and excessive response to evaluation were 0.858 and 0.814, respectively.

##### Autism spectrum quotient-Japanese version (AQ-J-10)

Based on a questionnaire originally developed by Baron-Cohen et al.^30^, the AQ-J-10 is a self-reported scale that measures autism-related traits among individuals with an IQ of at least 85. Since its reliability and validity have been confirmed^31^, the AQ-J-10 is commonly used to screen for ASD in Japan. Responses are provided on a 4-point Likert scale. For each item, the scale is split into halves, indicating high and low autism tendencies. If a participant selects one of the two options from the “high autism tendency” half, they are awarded 1 point, and if they select one of the two options from the “low autism tendency” half, they receive no points. The cut-off for the AQ-J-10 is seven points; that is, a score of seven points or more indicates a strong ASD tendencies.

### Statistical analysis

In the analysis, we first performed a simple tabulation for the attributes of the mothers and students. Specifically, we tabulated the distribution of grades and the number of male and female students (ratio). We conducted a simple aggregation of the mothers’ attributes and traits. We used matching data for the mother-student pairs. We calculated the average SOC score and its standard deviation for the students and mothers. Both SOCs were normally distributed. Next, we examined the students’ SOC by gender. We calculated the correlation coefficient between the subscale scores and total SOC score of the student and the SOC of the mother.

Additionally, we examined whether the average score on the School Membership Scale (by subscale), help-seeking style (by subscale), and PAS score, differed between male and female students. We examined the PAS (each subscale score) and the AQ-J-10 (total score) scores by gender, using the t-test. Furthermore, we calculated the Spearman correlation coefficient between each variable for both genders.

To examine gender differences in the factors related to a SOC, we performed a multiple regression analysis with SOC as the dependent variable. On calculating the correlation coefficient for each gender, we found a significant correlation for both genders for the School Membership Scale, help-seeking style, the PAS, interpersonal sensitivity, and the AQ-J-10. Therefore, we input these as explanatory variables. We used the SPSS 25.0 (IBM Japan, Ltd, Tokyo, Japan) for data analysis.

## Results

Among the students, 61.2% of the participants were female (Table 1). The average age of the mothers was 45.5 ± 4.1 years. The average SOC scores of the students and mothers were 39.9 ± 8.4 and 43.9 ± 6.6, respectively; the mothers’ scores were slightly higher than the average national SOC scores among Japanese women in their 40 s (42.2 ± 8.2)^32^.

**Table 1:** Attributes and characteristics of participants.

	n	(%)/SD
**Students**	134	
Male	52	(38.8)
Female	82	(61.2)
1st grade	67	(50.0)
2nd grade	67	(50.0)
SOC, mean (SD), range: 13–65	39.9	(8.4)
**Parents (Mother)**	134	
Age(SD)	45.5	(4.1)
**Employment**		
Regular-employment	34	(30.1)
Non-regular (part-time job)	60	(53.1)
Unemployed	19	(16.8)
**Marital status**		
Divorce/bereavement	5	(4.4)
Married	108	(94.7)
**Economic conditions**		
Poor/very poor	7	(6.2)
Average	41	(36.0)
Relatively rich/rich	66	(57.9)
SOC, mean (SD), range: 13–65	43.9	(6.6)

Missing values are excluded from the table.

*SOC* sense of coherence.

Table 2 shows the correlations between students’ and mothers’ SOC. The Pearson correlation coefficient between female students’ and their mothers’ SOC was 0.249 (*p* < 0.05). For the SOC subscales, the correlation between female students’ meaningfulness scores and mothers’ total SOC scores was 0.232 (*p* < 0.05); no other significant correlations were found. However, male students’ SOC scores were unrelated to their mothers’ SOC, and there was no correlation between male students’ SOC and their mothers’ responses for any of the other variables.

**Table 2:** SOC correlation of mother and child by gender.

	Male students n = 52				Female students n = 82			
	SOC total score	Subcomponents			SOC total score	Subcomponents		
		Meaningfulness	Comprehensibility	Manageability		Meaningfulness	Comprehensibility	Manageability
	r	r	r	r	r	r	r	r
**Mothers**								
SOC total score	–	–	–	–	0.249*	0.232*	–	–
SOC Subcomponents: Meaningfulness	–	–	–	–	–	–	–	–
Comprehensibility					–	–	–	–
Manageability	–	–	–	–	–	–	–	–

Variables that did not show significant differences were excluded.

*SOC* sense of coherence.

*p < 0.05.

Table 3 shows the average scores for each variable across male and female students. Results indicated that female students had higher scores for the excessive help-seeking style than male students (*p* = 0.011), as well as higher sensitivity to evaluation scores (*p* = 0.039) and monitoring scores for PAS (*p *= 0.039). Meanwhile, male students scored significantly higher on the psychological control subscale of the PAS (*p* = 0.001) and the AQ-J-10 (*p* = 0.039) compared with female students.

**Table 3:** Comparison of scale average by students' gender.

Variables	Male students n = 52		Female students n = 82		t test
	Average	SD	Average	SD	*p*-value
SOC total score (range 13–65)	40.5	8.8	39.5	8.2	0.489
**School membership scale**					
Acceptance by students (range 4–20)	15.4	3.2	15.7	3.3	0.626
Acceptance by teachers (range 5–25)	17.1	3.7	16.6	3.6	0.487
Sense of Belonging school (range 4–20)	15.9	3.1	16.0	3.4	0.915
**Help-seeking style**					
Excessive (range 4–28)	9.4	5.2	12.0	6.4	0.011
Avoidant (range 4–28)	11.8	6.0	11.1	6.4	0.549
**Parenting in adolescence scale**					
Acceptance (range 6–30)	23.5	6.3	24.2	5.1	0.502
Psychological control (range 6–30)	15.0	4.9	12.2	4.3	0.001
Monitoring (range 3–15)	11.6	2.6	12.9	1.9	0.003
**Interpersonal sensitivity scale**					
Sensitivity to evaluation (range 9–45)	29.9	7.1	32.8	8.7	0.039
Excessive response to evaluation (range 5–25)	14.5	4.3	15.9	4.6	0.067
Autism spectrum tendency (AQ-J-10) (range 0–10)	4.0	2.0	3.3	1.7	0.039

*SOC* sense of coherence.

Correlation coefficients for the student-related variables, stratified by gender, are shown in Table 4. The upper half presents the correlation coefficients for male students, while the lower half presents those for female students. In terms of correlations, male students' SOC total scores did not correlate significantly with their SOC total scores, in terms of excessive help-seeking style, acceptance and monitoring parenting score, but otherwise, both male and female students' SOC total scores were weakly to strongly significantly correlated with almost all variables (correlation coefficient r = − 0.753 to 0.627). In terms of correlations that differed by gender, on the AQ-J-10, male students’ scores were correlated with help-seeking styles (excessive: r = − 0.324, *p* < 0.01; avoidance: r = 0.398, *p* < 0.01) and scores on the School Membership Scale (acceptance from students: r =  − 0.519, *p* < 0.01; sense of belonging: r =  − 0.386, *p* < 0.01). However, female students’ AQ-J-10 scores were not correlated with any variable.

**Table 4:** Students’ correlation coefficient by gender.

		1	2	3	4	5	6	7	8	9	10	11	12
1	SOC	–	.576**	.436**	.612**	.013	−.395**	.235	− .424**	.138	− .636**	− .753**	− .395**
2	School membership: Accepted by students	.541**	–	.577**	.630**	.272*	− .345**	.436**	− .014	.320**	− .355**	− .241*	− .519**
3	School membership: Accepted by teacher	.365**	.444**	–	.311*	.264*	− .379**	.388**	− .184	.393**	− .223*	− .142	− .216
4	School membership: Sense of belonging	.627**	.776**	.467**	–	.329**	− .347**	.368**	− .057	.303**	− .396**	− .372**	− .386**
5	Help-seeking style: Excessive	.234*	.152	.148	.188	–	− .255	− .241	− .046	.342*	.223	.017	− .324**
6	Help-seeking style: Avoidant	− .475**	− .449**	− .129	− .466**	− .396**	–	− .241	.023	− .155	.246	.241	.398**
7	Parenting in Adolescence: Acceptance	.431**	.423**	.221	.127	.111	− .384**	–	− .411**	.395**	− .088	− .094	− .139
8	Parenting in Adolescence: Psychological control	− .220*	− .132	− .062	− .253	− .083	.242*	− .386**	–	− .134	.265	.359*	.067
9	Parenting in Adolescence: Monitoring	.349**	.237	.297*	.049	.242*	− .400**	.692**	− .194	–	.105	.052	− .107
10	Interpersonal Sensitivity: Sensitivity to evaluation	− .533**	− .231	− .143	− .336*	.069	.307**	− .169	.053	− .106	–	.787	.059
11	Interpersonal Sensitivity: Excessive response to evaluation	− .431**	.106	.322	.017	.097	.293**	− .134	− .068	− .125	.785**	–	.168
12	Autism spectrum tendency (AQ-J-10)	− .318**	− .058	− .120	− .134	− .176	.177	− .092	.090	− .151	.176	.123	–

*SOC* sense of coherence.

*p < 0.05, **p < 0.01.

In the multiple regression analysis (Table 5) using students’ SOC as the dependent variable, variables that were significant among female students included a sense of belonging to their school (β = 0.342, *p* = 0.012), sensitivity to evaluation (β =  − 0.265, *p* = 0.035), having ASD-related traits (β =  − 0.158, *p* = 0.049), and an avoidant help-seeking style (β =  − 0.186, *p* = 0.040). Among male students, the significant variables included a tendency toward an excessive response to evaluation (shocked and hurt by minor criticism; β =  − 0.442, *p* = 0.006), and feeling controlled by their parents (excessive psychological control by parents; β =  − 0.197, *p* = 0.049). The adjusted R^2^ scores were 0.715 and 0.530 for the male and female students, respectively. Thus, these traits appear to be related to a weak SOC.

**Table 5:** Factors related to students’ SOC (n = 134).

Variables	Male students (n = 52)				Female students (n = 82)			
	Multivariate				Multivariate			
	Coefficient β	*p*-value	standard error	95%cl	Coefficient β	*p*-value	standard error	95%cl
**School membership**								
Accepted by students	0.123	0.412	0.420	− 0.503 to 1.199	0.075	0.571	0.342	− 0.487 to 0.877
Accepted by teacher	0.144	0.177	0.246	− 0.160 to 0.839	− 0.049	0.609	− 0.049	− 0.541 to 0.319
Sense of belonging	0.160	0.195	0.347	− 0.246 to 1.163	0.342	0.012	0.324	0.188 –1.483
**Help-seeking style**								
Excessive	− 0.155	0.129	0.169	− 0.604 to 0.080	0.018	0.850	0.120	− 0.217 to 0.263
Avoidant	− 0.096	0.335	0.146	− 0.439 to 0.154	− 0.186	0.040	0.129	− 0.463 to − 0.053
**Parenting in adolescence**								
Acceptance	− 0.091	0.401	0.150	− 0.432 to 0.177	0.122	0.327	0.201	− 0.202 to 0.599
Psychological control	− 0.197	0.049	0.174	− 0.700 to − 0.002	− 0.089	0.321	0.170	− 0.509 to 0.169
Monitoring	0.126	0.191	0.323	− 0.225 to 1.084	0.014	0.903	0.484	− 0.906 to 1.024
**Interpersonal sensitivity**								
Sensitivity to evaluation	− 0.104	0.473	0.178	− 0.491 to 0.232	− 0.265	0.035	0.126	− 0.523 to − 0.020
Excessive response to evaluation	− 0.422	0.006	0.290	− 1.439 to − 0.262	0.008	0.955	0.241	− 0.468 to 0.495
Autism spectrum tendency (AQ-J-10)	− 0.154	0.122	0.442	− 1.595 to 0.196	− 0.158	0.049	0.399	− 1.574 to − 0.014
Total adjusted R^2^	0.715				0.530			

Missing values have been removed.

*SOC* sense of coherence.

## Discussion

### Relationship between children’s and mothers’ SOC

To our knowledge, this is the first study to examine the relationship between early adolescent students’ and their mothers’ SOC scores. Although we found a weak correlation between mothers and daughters in terms of parent–child SOC, this relationship was absent between mothers and sons, as we found no correlation between male students’ and their mothers’ SOC scores. This result may indicate that a SOC is not mutually shared among a family’s members. This study used a cross-sectional design, and therefore, causal relationships could not be determined; however, it is plausible that adolescents’ SOC may be affected by relationships and experiences occurring outside the family. Although not shown in the tables, the mothers’ high economic status and high educational background were not related to their children’s SOC. Interestingly, previous studies have found an association between a SOC and high socioeconomic status and educational background^6,32^; however, we could not confirm this finding in our sample. Togari et al.^33^, examined a SOC in mothers of high school students and found that mothers’ SOC affected their children’s SOC. Thus, it is unclear whether the difference between the present and previous results is because of age difference between the samples, differences between private and public schools (i.e., high versus free tuition), or the location of the schools.

With regard to the association of a SOC between mothers and daughters, Egami and Nakata^34^ reported gender differences in children’s affinity for their parents, and this affinity was particularly strong in mother-daughter relationships. High levels of closeness between mothers and their daughters have long been reported in Japan^35^, and situations where the father is commonly absent are notable in this regard^21^. Further, male junior high school students have close psychological proximity with their fathers, who are their primary role models^34^. Consequently, the SOC of male students may be strongly related to that of their fathers. Thus, future research on the association between children’s and parents’ SOC should also include fathers.

### Students’ scores and gender differences

As mentioned, female students exhibited higher scores for help-seeking behavior and sensitivity to evaluation, compared with male students (Table 3). However, as shown in Table 4, there was a positive correlation between excessive help-seeking behavior in female students and their scores on the School Membership Scale. Previous research suggests that, according to students, trust and a sense of unity are essential for seeking help; therefore, seeking help in school suggests that students view their school as a safe place^36^. However, this result was not found in male students. This study showed that both male and female students who do not seek help, lack a sense of belonging to their school and do not feel accepted by their teachers. This suggests that teachers and families should pay closer attention to students who do not express their difficulties or emotions.

Furthermore, our findings showed that male students exhibited higher ASD-related traits (via the AQ-J-10) than female students. Research indicates that children who have higher ASD-related traits usually do not seek help^20^, even if they are experiencing difficulties, and feel that they are not accepted by other students, and therefore, do not have a safe space in the school. However, no variables were correlated with ASD-related traits among the female students, despite the fact that the association was found for men; this may suggest, paradoxically, that women with high ASD-related traits may find it challenging to seek help, because they believe they are not experiencing much difficulty. In other words, a female student with high ASD-related traits—and her teacher—may first realize that she is having difficulties only after the student develops a secondary disorder, such as depression or an adaptation disorder. Therefore, teachers and school nurses should carefully assess students with ASD-like traits.

We also obtained results on the correlations among children’s characteristics and interpreted them. However, it is clear that concerns regarding the theoretical justification of these findings still remain. One possible reason for this is that very few studies have examined the association between ASD tendencies and other characteristics in junior high school students attending regular schools. From the perspective of an enhanced SOC and to ensure proper development in adolescents, it is necessary to accumulate more research on the characteristics of adolescents in this age group.

### Gender differences among SOC-related factors

The results of the multiple regression analysis showed that, among female students, sensitivity to evaluation (i.e., worrying about how others see them), ASD-related traits, and an avoidant help-seeking style were significantly and negatively associated with a SOC. Importantly, adolescent female students often show a strong tendency to care about others’ opinions^37^, which was confirmed in this study. Although it is natural to be apprehensive about the opinions of others, there is a need to educate female students, both at school and at home, that one should not be overly concerned in this regard. Additionally, given that female students build relationships in consideration of their surroundings and the harmony therein, known in Japan as “reading the context”^37^, it is possible that female students with ASD-related traits commonly experience severe difficulties in friendships at school. Indeed, in this study, female students tended to seek help more when compared to male students. However, children with ASD often do not seek help when needed^19,20^. Moreover, female students were more likely to seek help than male students; however, female students who do not seek help in need, are less likely to be noticed by their teachers or parents. Furthermore, female students who are likely to have ASD, may not know how to seek help or who to seek help from, and might not even understand that they are facing difficulties. Therefore, it is important for teachers and parents to actively ask such female students, “Are you experiencing any difficulty?” Furthermore, teachers and parents should secure a place for them where they can communicate and express themselves freely.

For male students, there was a significant negative association between a SOC and a strong tendency toward excessive response to evaluation (shocked or hurt by minor criticism). Previous studies have reported that male students greatly value their relationships with peers and seniors^38^. Furthermore, Bando and Mori^39^ found that male students experience more social pressure, particularly when exposed to others’ opinions. Thus, attention should be paid to male students, especially those who are sensitive to the reactions of others, and teachers should closely observe how they respond to interactions with peers. In this study, among male students, the feeling of being controlled by their parents (excessive parental control) was correlated with a weak SOC. Excessive attention from mothers, typified by statements such as “Have you finished your homework?” or “Stop using your smartphone” may be interpreted by male adolescents as “You are a helpless and useless child.” Relevant literature states that adolescents who rebel against attentive parents are likely to be healthier^37^. Thus, we suggest that schoolteachers should monitor obedient or quiet male students who have attentive mothers, as well as their mental health status. While mothers need to be attentive to their sons, it is also crucial to educate mothers on how to interact with their sons at home—for example, informing them that it is better not to repeatedly give detailed instructions to boys.

Regarding ASD-related traits, male students’ scores were higher than those of female students; however, there was no association with a SOC. Social development in adolescence occurs more rapidly in female than in male students^40^. Given that male students tend to be more immature compared to female students of the same age, it is possible that male students with ASD are not bothered by their communication skills, because they are part of groups having members with similar characteristics. However, they may face difficulties upon reaching a stage that requires social communication, such as high school and college. Thus, proper career guidance—based on personality characteristics—is important for preventing secondary disorders, such as depression.

### Limitations

A major limitation of this study is that we only surveyed one school in Japan. This is because our questionnaire—which was a parent–child survey—contained sensitive content, and therefore, it was difficult to gain cooperation from schools. The school nurses and teachers in the target school were eager to care for the students. Additionally, it was a public school with several academic achievements and where many students came from families with a high socioeconomic status; therefore, the present results should be generalized with caution. Another limitation was the low response rate. Ethical considerations were required because the sample comprised junior high school students—specifically, we were required to acquire written consent from the parents of each student. Thus, the submission rate of the written consent forms was the response rate. Failure to adequately explain the study to parents—because of time constraints—could have negatively affected the response rate. A higher number of participants would have allowed us to examine additional variables. Furthermore, the parents (mothers) who consented and participated in the study may have been a group of mothers with a strong SOC, who were interested and enthusiastic about their children's care and school life. This can be seen from the fact that the mothers' SOC scores were higher than those of their peers in the national sample. This population characteristic needs to be understood and considered when interpreting the results.

In this study, we only included mothers, as they are considered to be the parent primarily involved in child-rearing in Japan. Although we found an association between female students’ and their mothers’ SOC, it is possible that a similar association between male students’ and their fathers’ SOC could have been observed, if fathers had been included in this investigation. To enable proper physical and psychological development in children, it may be necessary to provide fathers with recommendations on how to behave at home and interact with their children. Thus, further research regarding the role of fathers in adolescent SOC formation is necessary and warranted.

## Conclusion

The SOC of female junior high school students was slightly correlated with their mothers’ SOC. However, there was no correlation between male students’ SOC and mother-related variables. This may be due to the high affinity between mothers and daughters at home, in Japan. In the future, it will be important to consider the relationship between boys’ and their fathers’ SOC. Furthermore, although there were no gender differences in the SOC scores, such differences were found in other variables, such as ASD-related traits being higher in male students than in female students, and the latter seeking help more than the former. The multiple regression analysis showed that, among female students, factors such as ASD-related traits and an evasive help-seeking style were negatively related to a SOC. Given that many female students are usually active in seeking help, teachers and parents need to pay attention to quiet female students who do not seek help. At school, establishing a system of counseling and outreach can make it easier for students to receive help.

Excessive response to evaluation and a strong sense of parental control were negatively related to a SOC among male students. Thus, persistent attention from mothers may reduce their sons’ SOC. It is important to observe male students who receive too much attention from their mothers and are quiet and obedient. Ultimately, the school and home environments both have different influences on adolescent boys and girls; thus, it is necessary to create environments that accommodate these gender differences within schools and homes.

## Data Availability

The data in this study is a questionnaire survey of parents and children in junior high school and is very sensitive, including information on autism regarding legal minors. The schools surveyed are not allowed to disclose raw data, even anonymously.

## References

[1] Antonovsky A (1979). Health, Stress, and Coping: New Perspectives on Mental and Physical Well-Being.

[2] Antonovsky A (1987). Unraveling the Mystery of Health: How People Manage Stress and Stay Well.

[3] García-Moya I, Moreno C, Jiménez-Iglesias A (2013). Understanding the joint effects of family and other developmental contexts on the sense of coherence (SOC): A person-focused analysis using the classification tree. J. Adolesc..

[4] García-Moya I, Rivera F, Moreno C, Lindström B, Jiménez-Iglesias A (2012). Analysis of the importance of family in the development of a sense of coherence during adolescence. Scand. J. Pub. Health.

[5] Merakou K (2013). Sense of coherence in people with and without type 2 diabetes mellitus: An observational study from Greece. Ment. Health Fam. Med..

[6] Tsuno YS, Yamazaki Y (2012). Relationships among sense of coherence, resources, and mental health in urban and rural residents in Japan. BMC Publ. Health.

[7] Omiya T, Yamazaki Y (2017). Positive change and sense of coherence in Japanese mothers of children with congenital appearance malformation. Health Psychol. Open.

[8] Omiya T, Dumais N (2017). Difficulties and Coping Strategies Experienced by Employed People with HIV. Japan: A Qualitative Study Comparing High and Low Sense of Coherence Groups in HIV/AIDS—Contemporary Challenges.

[9] Sairenchi T (2011). Sense of coherence as a predictor of onset of depression among Japanese workers: A cohort study. BMC Pub. Health.

[10] Berg JE (2010). Death by suicide long after electroconvulsive therapy. Is the sense of coherence test of Antonovsky a predictor of mortality from depression?. Ment. Illn..

[11] Feldman DB, Einav M, Margalit M (2018). Does family cohesion predict children’s effort? The mediating roles of sense of coherence, hope, and loneliness. J. Psychol..

[12] Antonovsky A (1996). The salutogenic model as a theory to guide health promotion. Health Promot. Int..

[13] Omiya T, Deguchi N, Togari T, Yamazaki Y (2020). Factors influencing sense of coherence: Family relationships, high school life and autism spectrum tendency. Children (Basel).

[14] Boyce P, Parker G (1989). Development of a scale to measure interpersonal sensitivity. Aust. N. Z. J. Psychiatr..

[15] Ishizu K, Ambo H (2007). Over-adapted tendency and childhood depression: A study of Japanese junior-high school students. Ann. Rep. Grad. Sch. Educ. (Tohoku Univ., 2007).

[16] Soma S (2005). Psychology of a child who tries too hard (improves motivation to study). Child Study.

[17] Baron-Cohen S (2009). Prevalence of autism-spectrum conditions: UK school-based population study. Br. J. Psychiatr..

[18] Kamio Y (2013). Quantitative autistic traits ascertained in a national survey of 22 529 Japanese schoolchildren. Acta Psychiatr. Scand..

[19] Kamio Y, Inada N, Koyama T (2013). A nationwide survey on quality of life and associated factors of adults with high-functioning autism spectrum disorders. Autism.

[20] Kamio Y (2013). Psychiatric issues of children and adults with autism spectrum disorders who remain undiagnosed. Seishin Shinkeigaku Zasshi.

[21] Shoji J, Taniguchi W (1999). Father’s absence and mental problems of children. Jpn. J. Res. Fam. Nurs..

[22] Ekino M (2013). A study of modern fathers in clinical psychology. Bull. Hiroshima Bunkyo Womens Univ..

[23] Togari T, Yamazaki Y (2005). Examination of the reliability and factor validity of 13-item five-point version sense of coherence scale. Jpn. J. Health Hum. Ecol..

[24] Goodenow C (1993). The psychological sense of school membership among adolescents: Scale development and educational correlates. Psychol. Sch..

[25] Togari T, Sato M, Yamazaki Y, Otemori R (2011). The development of a Japanese 13-item version of psychological sense of school membership scale for Japanese urban high school students. J. Sch. Health.

[26] Nagai S (2013). Development of a scale for measuring help-seeking styles. Jpn. J. Educ. Psychol..

[27] Utsumi S (2013). Development of the parenting in adolescence scale (PAS). Shinrigaku Kenkyu.

[28] Schaefer ES (1965). Children’s reports of parental behavior: An inventory. Child Dev..

[29] Muranaka M, Yamakawa I, Sakamoto S (2018). Development of the interpersonal sensitivity/privileged self scale: The measurement of a psychological characteristic related to “modern-type depression”. Jpn. J. Psychol..

[30] Baron-Cohen S, Wheelwright S, Skinner R, Martin J, Clubley E (2001). The autism-spectrum quotient (AQ): Evidence from asperger syndrome/high-functioning autism, males and females, scientists and mathematicians. J. Autism Dev. Disord..

[31] Kurita H, Koyama T, Osada H (2005). Autism-spectrum quotient-Japanese version and its short forms for screening normally intelligent persons with pervasive developmental disorders. Psychiatr. Clin. Neurosci..

[32] Togari T (2015). Nationally representative score of the Japanese language version of the 13-item 7-point Sense of coherence scale. Nihon Koshu Eisei Zasshi.

[33] Togari T (2012). Sense of coherence in mothers and children, family relationships and participation in decision-making at home: An analysis based on Japanese parent-child pair data. Health Promot. Int..

[34] Egami S, Nakata S (2018). Determinant and outcome of mother-daughter connected relationships. Bull. Fac. Educ. Ehime Univ..

[35] Kitamura K, Muto T (2003). Adult mother-daughter relationship and mother’s psychological well-being. Shinrigaku Kenkyu.

[36] Ijadi-Maghsoodi R (2018). Voices from minority youth on help-seeking and barriers to mental health services: Partnering with school-based health centers. Ethn. Dis..

[37] Horii T (2002). Developmental changes of negative self-awareness in interpersonal relationships during adolescence (Second Report). Bull. Yamagata Univ. (Soc. Sci.).

[38] Kogawa T, Nishizawa Y (2011). Relation between parent-child relationship and lifestyle based on a family diagnostic test of junior high school students. Pediatr. Health Res..

[39] Bando K, Mori Y (2013). Factors affecting eating disorders during puberty. J. Home Econ. Jpn.

[40] Katou H, Ota M, Matsushima M, Mitsui Y (2014). A development of thinking in puberty and its influences on junior high school students’ self-esteem and interpersonal relationships. Ann. Rep. Res. Clin. Cent. Child Develop..

